# Crosstalk between KRAS, SRC and YAP Signaling in Pancreatic Cancer: Interactions Leading to Aggressive Disease and Drug Resistance

**DOI:** 10.3390/cancers13205126

**Published:** 2021-10-13

**Authors:** Enrique Rozengurt, Guido Eibl

**Affiliations:** 1 Department of Medicine, David Geffen School of Medicine at UCLA, Los Angeles, CA 90095, USA; 2 Department of Surgery, David Geffen School of Medicine at UCLA, Los Angeles, CA 90095, USA; Geibl@mednet.ucla.edu

**Keywords:** pancreatic cancer, KRAS dimerization, YAP/TAZ, Hippo pathway, Src family kinases, gene regulatory networks

## Abstract

**Simple Summary:**

Pancreatic ductal adenocarcinoma (PDAC), the most common form of pancreatic cancer, remains one of the most lethal diseases. Current evidence, discussed in this article, implicates an interplay between the oncogene KRAS, the transcriptional co-activator YES1-Associated Protein (YAP) and the proto-oncogenes of the Src family kinases (SFK) in the pathogenesis of PDAC and consequently, they represent potential targets for therapeutic intervention. Here, we focus on recent mechanistic and translational studies that identify a complex crosstalk between KRAS, YAP and SFK in PDAC initiation and maintenance. Additionally, we discuss strategies for targeting these crucial signaling nodes and the feedback loops emanating from them, with especial consideration to preventing the development of drug resistance.

**Abstract:**

Pancreatic ductal adenocarcinoma (PDAC), the predominant form of pancreatic cancer, remains a devastating disease. The purpose of this review is to highlight recent literature on mechanistic and translational developments that advance our understanding of a complex crosstalk between KRAS, YAP and Src tyrosine kinase family (SFK) in PDAC development and maintenance. We discuss recent studies indicating the importance of RAS dimerization in signal transduction and new findings showing that the potent pro-oncogenic members of the SFK phosphorylate and inhibit RAS function. These surprising findings imply that RAS may not play a crucial role in maintaining certain subtypes of PDAC. In support of this interpretation, current evidence indicates that the survival of the basal-like subtype of PDAC is less dependent on RAS but relies, at least in part, on the activity of YAP/TAZ. Based on current evidence, we propose that SFK propels PDAC cells to a state of high metastasis, epithelial-mesenchymal transition (EMT) and reduced dependence on KRAS signaling, salient features of the aggressive basal-like/squamous subtype of PDAC. Strategies for PDAC treatment should consider the opposite effects of tyrosine phosphorylation on KRAS and SFK/YAP in the design of drug combinations that target these novel crosstalk mechanisms and overcome drug resistance.

## 1. Introduction

One of the deadliest types of cancer has been and still is pancreatic ductal adenocarcinoma (PDAC), the predominant form of pancreatic cancer (~90%). A recent report of the American Cancer Society estimates 60,430 new cases (28,480 females and 31,950 males) of pancreatic cancer in the year 2021. An estimated 48,220 patients (22,950 female and 25,270 male patients) will succumb to this disease, putting pancreatic cancer as the third leading cause of cancer mortality in the USA [1]. Moreover, PDAC is anticipated to exceed the deaths from colorectal cancer by 2030, thus becoming the second leading cause of cancer-related deaths before 2030 [2]. Indeed, only a minority of patients with PDAC (15–20%) are eligible for surgical resection due to early local and distant spread. Furthermore, the benefit in prolonging survival by current neo-adjuvant and adjuvant chemotherapeutic regimens is modest and recurrences occur often [3,4]. Consequently, the 5-year survival rate in PDAC has stayed at around 9%. Clearly, these sobering statistics provide strong impetus to search for novel strategies for early diagnosis and for identifying new targets, agents and combinatorial approaches for prevention and therapeutic intervention. The elucidation of the molecular mechanisms driving PDAC growth and dissemination will be of crucial significance to guide the discovery of novel biomarkers, targets and agents for therapeutic intervention. The purpose of this review is to highlight recent mechanistic and translational developments that in the opinion of the authors advance our understanding of the complex interplay between KRAS, Yes-Associated Protein (YAP) and Src tyrosine kinase family (SFK) in PDAC development and in designing novel combinatorial therapies. Multiple other therapies for the treatment of PDAC are under development [5], including targeting epigenetic control [6], the extracellular matrix and the immune system [7] but their discussion is beyond the scope of this article. 

## 2. KRAS and PDAC

Extensive research in both humans and mice have corroborated the significance of *KRAS* (encoding KRAS) mutations in the initiation and maintenance of PDAC [8,9]. The RAS proteins cycle between a GTP-bound active state and a GDP-bound inactive state [10], characterized by different conformations [8]. This cycle is regulated by GTP exchange factors (RAS GEFs), including son of sevenless homolog 1 (SOS1), which promote the GTP-bound state and GTPase activating proteins (RAS GAPs) that accelerate the hydrolysis of GTP returning RAS to the GDP-bound state [11]. In normal quiescent cells, RAS is predominantly GDP-bound and inactive. Stimulation of receptor tyrosine kinases (RTKs), G protein-coupled receptors (GPCRs), adhesive receptors of the integrin family and other cell-surface receptors (e.g., cytokines), induces a rapid and transient increase in the concentration of RAS-GTP at the inner leaflet of the cell membrane. Active RAS engages effector proteins that then regulate intracellular signaling pathways that promote a mitogenic response, the best characterized of which are the Raf serine/threonine kinases/mitogen activated protein (MAP) kinase kinase (MEK)/extracellular signal-regulated kinase (ERK) and phosphatidylinositol 3-kinase (PI3K)/protein kinase B (AKT)/mechanistic target of rapamycin (mTOR) pathways. Ras effectors contain a RAS binding domain (RBD) that binds primarily to RAS-GTP, leading to proliferative [9] and apoptotic [12] cellular responses [11]. 

Multiple genomic studies showed that approximately 90% of PDACs harbor mutations in *KRAS* [13,14,15], a result further corroborated by a recent major targeted genomic profile analyses of 3594 primary and metastatic PDAC samples from an international cohort [16]. Most *KRAS* mutations in PDAC occur at position G12, of which the single amino acid replacement glycine to aspartic (G12D) is the prevalent substitution. KRAS mutations in position G12 prevent interaction between KRAS and GAPs [10], thereby leading to lasting KRAS activation and stimulation of downstream signaling pathways [17]. KRAS^G12C^, an uncommon mutation in PDAC (2–3% of *KRAS* mutants in PDAC), can be inhibited by drugs (e.g., AMG 510) targeting Cys-12 [18]. In contrast, KRAS^G12D^ is not a direct target for any existing drug. Pancreatic cancers without *KRAS* mutations (about 10% of PDAC) show wild type RAS activation via upstream signaling through receptor tyrosine kinases, including epidermal growth factor receptor (EGFR), which is also required in KRAS^G12D^-driven PDAC [16,19,20]. Furthermore, a small number (3%) of PDAC patients harbor oncogenic activation of the RAF serine/threonine kinase B-RAF [15,16].

In most cases, PDAC arises through a stepwise progression from precursor lesions [21], the most prominent of which are pancreatic intraepithelial neoplasia (PanINs). In this setting, an activating *KRAS* mutation is an early and initiating event detected in PanIN lesions [22]. However, a subgroup of PDAC (as much as 30%) appears to develop within a short period by means of major chromosomal rearrangements via chromothripsis, instead of gradual progression and accumulation of individual genetic mutations [23].

Genetically engineered mouse models of PDAC have corroborated a critical role of KRAS in PDAC [24,25]. In a widely used model (known as KC), mutated *Kras* (mouse ortholog of human *KRAS*) is expressed from its endogenous locus by crossing *LoxP-*Stop*-LoxP* (*LSL*)-*Kras^G12D^* mice with *PDX-1-Cre* or *p48-Cre* mice. This mouse model displays similar histopathologic features to the human PDAC, including the development and progression of PanINs [24]. However, there is increasing recognition that oncogenic *Kras* promotes different lesions depending on whether is expressed in acinar or ductal cells [26,27,28,29]. Specifically, acinar cell-derived tumors proceed through low-grade PanINs whereas ductal cell-derived tumors give rise to high-grade PanIN and invasive PDAC bypassing low-grade PanINs [28]. 

Although mutations in *KRAS* is an early and essential step in most cases of PDAC, it is insufficient to stimulate development of invasive PDAC. Activation/inactivation of other pathways by additional mutations (e.g., in tumor suppressor genes, including *CDKN2A*, *TP53* and *SMAD4*) or environmental stimuli, including obesity and chronic inflammation are required for the promotion of high-grade PanIN and PDAC [30]. Inactivation of *CDKN2A* is an early event in PDAC development whereas inactivation of *TP53* and *SMAD4* are late events in the multi-step model of PDAC progression [31]. *TP53* often undergoes missense mutation resulting in gain of function rather than loss of expression [32]. Large-scale studies have uncovered roles for additional genetic and epigenetic alterations, including those encoding SWI/SNF-mediated chromatin remodeling complexes [33] and histone modification enzymes and for polyploidy and chromothripsis as factors contributing to pancreatic cancer biology and progression (reviewed in [34]). In the setting of PDAC and other cancers, the molecular mechanisms by which KRAS functions at the cell membrane to activate its effectors [35] are of intense interest and translational importance for drug discovery that remain incompletely understood. Another area of major significance is the dependency on oncogenic *KRAS* of different molecular subtypes of PDAC and the identification of mechanisms that bypass KRAS signaling. These represent major foci of this article, which are discussed in subsequent sections.

### 2.1. KRAS Dimerization in Signal Transduction

**Figure 1:**
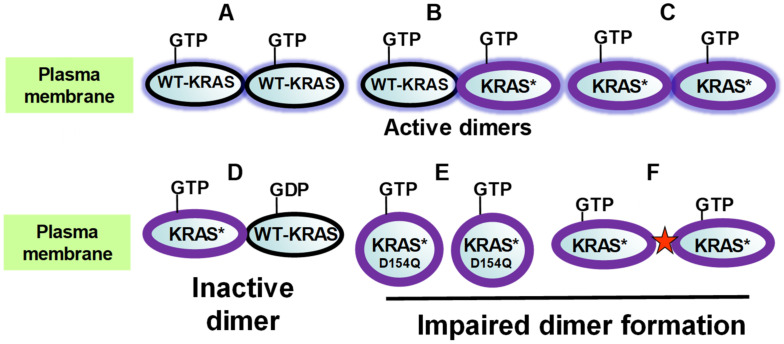
Importance of dimerization in RAS biology. (**A**–**C**): Dimerization of wild type (WT)-KRAS or KRAS^G12D^ (indicated by KRAS*) in their GTP-bound state is necessary for KRAS signaling. (**D**): WT-KRAS in the GDP-bound form signaling inactive dimers with KRAS* thus explaining the tumor suppressive effects of the WT allele in cells harboring one copy of WT and one copy of mutant *KRAS*. Activation of the WT-KRAS to the GTP-bound state in response to growth factors, GPCR agonists, integrin engagement and/or inflammatory mediators restores the formation of active dimers. (**E**,**F**): Inhibition of dimerization by mutation (D154Q) or a monobody (red star) blocks KRAS signaling, thus providing strong evidence for the dimerization hypothesis. Please also see the main text for details.

Growing evidence supports the notion that RAS proteins form dimers or oligomers, including transient nanoclusters, at the inner leaflet of cell membranes and that RAS dimerization plays a major role in signal transduction [11], as schematically illustrated in Figure 1. Several lines of evidence support the dimerization hypothesis. For example, using a tetracycline-regulated expression system and high-resolution microscopy, Nan et al. [36] concluded that KRAS^G12D^ formed dimers as its concentration increases in cells. Downstream signaling, including extracellular signal-regulated kinase (ERK) phosphorylation was activated when Kras^G12D^ concentration reached a threshold that coincided with images interpreted as indicative of dimerization [36]. In addition, forced dimerization of Kras^G12D^ triggered ERK activation, supporting the conclusion that RAS dimerization is a critical step leading to downstream signaling. 

Recently, Ambrogio et al. identified a mutation in the allosteric lobe of Kras (D154Q) that blocked dimer formation and prevented transformation by Kras^G12D^ [37]. The mutation did not affect other aspects of RAS function, including cycling between GTP and GDP bound states. These results provided strong support for the notion that dimerization of oncogenic KRAS is necessary for signal transduction [37]. Furthermore, a genetically engineered small antibody mimetic targeting the putative interface of the RAS dimer inhibited dimerization, downstream signaling, and transformation [38,39]. A recent study, using chemical and biophysical approaches with different KRAS forms provided additional evidence in favor of RAS dimerization and proposed a structural model of the interacting regions in the RAS dimer, an important step for identifying new drugs that interfere with dimerization and RAS function [40]. Figure 1 summarizes these concepts.

Importantly, RAS dimerization fits well with the known requirement for dimerization and trans-phosphorylation for activation of the members of the RAF family [41,42,43]. Specifically, binding of C-RAF and B-RAF to KRAS-GTP or KRAS^G12D^ dimers via their RBD (Ras binding domain) and CRD (cysteine-rich domain), facilitates RAF membrane localization, dimerization, release of auto-inhibition and induction of multiple phosphorylation/de-phosphorylation events that lead to RAF activation [43,44,45]. In the RAF dimer, one protomer (B-RAF) acts as an allosteric activator of the other protomer (C-RAF). RAF dimerization is of critical importance in the mechanisms leading to drug resistance [46]. Collectively, these studies emphasize that RAS dimerization is a critical step in RAS signaling, though methodological inconsistencies also remain [47]. 

RAS dimerization also provides a cogent model to explain the selective pressure for losing the wild type allele in PDAC and other cancers [48] and offers an attractive new route to discover drugs that prevent RAS function. Figure 1 shows some of the salient features of wild type and mutant KRAS dimerization. Specifically, the wild type isoform of KRAS is tumor suppressive in its GDP bound state because it forms inactive dimers with the oncogenic KRAS, thereby inhibiting its function. Conversion of the wild type KRAS to its GTP-bound state in response to extracellular signals, including growth factors, GPCR agonists and integrins enables it to form functionally active dimers with the mutated KRAS. This model also could explain the common finding that human pancreatic cancer cells in growth factor-depleted medium exhibit very low ERK activity despite harboring mutated *KRAS* [49], which is constitutively active but suppressed by the unstimulated wild type allele. Growth factor stimulation of the cells induces the GTP-bound state of the wild type KRAS, restores the formation of signaling productive dimers, and thus induces robust ERK activation (Figure 1). 

The majority of PDACs had allelic imbalances causing increased *Kras^G12D^* gene dosage, which occurs early in PDAC evolution, increases metastatic potential and appears to be contingent to the loss of tumor suppressive genes, including *Cdkn2a* and *Tp53* [50]. Interestingly, gene dosage gain of *KRAS^G12D^* was associated with loss of wild-type *KRAS* in PDAC. A previous study showed that mice with pancreas specific *Cdkn2a* inactivation and *Kras^G12D^* activation developed the full spectrum of PanIN lesions and PDAC with metastasis [51]. Interestingly, the *Kras^WT^* allele was lost during the progression from primary tumors to metastases in the pancreas from *Cdkn2a^Null^-Kras^G12D^* mice [51]. Other studies also support the concept that the wild type allele of *Kras^G12D^* functions as a tumor suppressor [48]. While, as mentioned above, wild type Kras suppresses the oncogenic activity of Kras^G12D^, a dimerization-deficient wild-type Kras mutant did not inhibit the oncogenic effects of Kras^G12D^, implying that the tumor suppressive effects of the wild type Kras is exerted via formation of an unproductive dimer with Kras^G12D^ [37]. Thus, RAS dimerization, as illustrated in Figure 1, offers a plausible molecular mechanism that explains both the selective pressure that favors increased Kras^G12D^ gene dosage, as well as elimination of the wild type allele. 

It should be pointed out that dimerization is thought to occur between the same RAS isoforms. Specifically, wild type RAS of the same isoform in its GDP bound state inhibits the function of the mutated isoform whereas overexpression or stimulation of the protein products of the two non-mutated, wild type *RAS* genes (e.g., *HRAS* and *NRAS* in a *KRAS*-mutated cancer) are tumor promoting in RAS-mutated tumors (reviewed in [52]). Accordingly, increased expression of NRAS or high expression of autocrine EGFR ligands, including amphiregulin [53] that activate RAS isoforms are associated with unfavorable prognosis in PDAC.

Most studies concerning the role of KRAS dimerization on downstream signaling focused on the RAF/MEK/ERK cascade. It is unclear whether KRAS-GTP dimers are also necessary for the activation of other RAS effectors, including PI3K. Furthermore, most studies did not consider additional complexities caused by other post-translational modifications of the RAS proteins (reviewed in [11] and references therein), including phosphorylation. In the following section, we will discuss recent studies that focused on the impact of direct tyrosine phosphorylation on KRAS cycling between active and inactive states and on the engagement of KRAS with downstream effectors.

### 2.2. Regulation of KRAS Function by Tyrosine Phosphorylation: Contrasting Roles of SFK and SHP2 

There is increasing interest in elucidating KRAS regulation by direct tyrosine phosphorylation, primarily by members of the structurally related non-receptor Src family of tyrosine kinases (SFKs). The SFK comprises 12 members, (Src, Fyn, Yes, Yrk, Lyn, Hck, Fgr, Blk, Lck, Brk, Srm, and Frk) three of which, Src, Fyn, and Yes, are expressed prominently in many cell types, including human pancreatic cancer cell lines [54], while other members have a more restricted expression pattern, especially to cells of hematopoietic origin [55]. 

Recent studies using in vitro kinase reactions and expression of KRAS or KRAS mutants in cells demonstrated that Src phosphorylates KRAS on Tyr^32^, in the switch region I and Tyr^64^, in the switch region II [56]. These regions of KRAS, located in the catalytic lobe participate in GDP-GTP exchange and in the binding of effectors (e.g., RAF) and GAPs [57]. Src-mediated KRAS phosphorylation on Tyr^32^ and Tyr^64^ induces conformational changes that inhibit KRAS function, including its ability to stimulate downstream RAF/MEK/ERK signaling [56,57,58]. In addition, tyrosine phosphorylation of KRAS inhibits its membrane localization [59], suggesting that phosphorylation could decrease KRAS signaling, at least in part, by reducing dimer formation at the membrane. The precise impact of tyrosine phosphorylation of KRAS on its dimerization warrants further experimental work.

**Figure 2:**
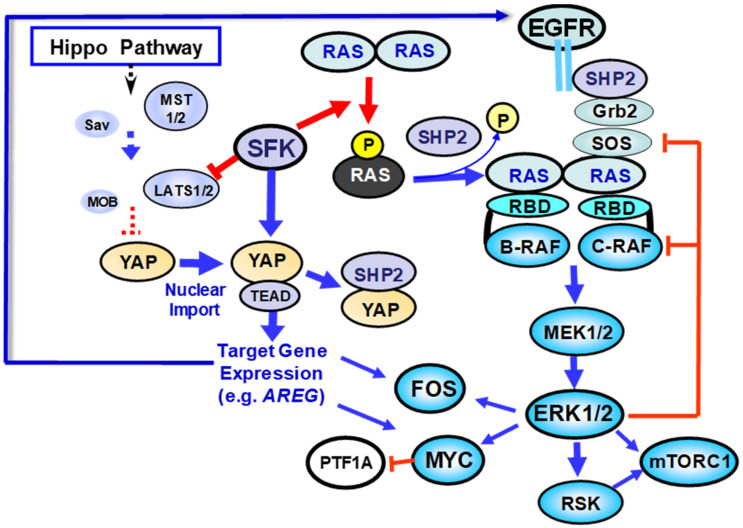
Opposite effects of SFK-mediated phosphorylation on KRAS and YAP function. SFK-mediated KRAS phosphorylation on Tyr^32^ and Tyr^64^ inhibits KRAS signaling by decreasing affinity for the RBD of effectors (e.g., RAF). Concomitantly, SFK activates YAP via Hippo-dependent pathway by repression of LATS1/2 and/or Hippo-independent direct tyrosine phosphorylation, including phosphorylation of YAP at Tyr^357^. SFK-mediated YAP activation leads to nuclear import and complex formation with the transcription factors of the TEAD family, leading to increase expression of many genes that regulate cell survival, proliferation, EMT, mitosis, adaptation to hypoxia and release of factors that induce autocrine/paracrine stimulation of EGFR, including amphyregulin (AREG). The tyrosine phosphatase SHP2 opposes SFK-mediated phosphorylation, thereby reactivating KRAS but inactivating YAP. RAS dimers lead to RAF/MEK/ERK activation. In turn, ERK phosphorylates multiple substrates, leading to increased expression of c-FOS, stabilization of c-MYC and activation of the p90 ribosomal S6 kinases (RSK). MYC inhibits the transcription factor PTF-1A, which controls the expansion of pancreatic progenitor cells and the acinar cell lineage. ERK and RSK lead to mTORC1 activation (omitted in the scheme for clarity). ERK also phosphorylates and inhibits upstream elements of the pathway (e.g., RAF and SOS), thus mediating feedback regulation that fine-tunes the activity of the pathway. Blue lines represent stimulation. Red lines represent inhibition. Please also see the main text for additional details.

The oncogenic Src homology-2 (SH2) domain-containing phosphatase 2 (SHP2), encoded by *PTPN11*, reverses the tyrosine phosphorylation of KRAS on Tyr^32^ and Tyr^64^ [60]. A subsequent study identified Protein Tyrosine Phosphatase Non-Receptor Type 2 (PTPN2) as another phosphatase that dephosphorylates KRAS and regulates its downstream signaling [59]. Collectively, these results support the notion that KRAS phosphorylation on Tyr^32^ and Tyr^64^ inhibits KRAS function [56,57,58,60]. These novel and surprising findings change the conventional paradigm of RAS regulation and imply that SFK-mediated tyrosine phosphorylation of KRAS restricts KRAS function [56], as depicted in Figure 2. 

A corollary of this notion is that SFK inhibitors could induce KRAS hyper-activation leading to RAF/MEK/ERK signaling, thus explaining their lack of antitumor efficacy in the clinic, a point that we will revisit in a subsequent section. While tyrosine phosphorylation of RAS proteins inhibited interaction with downstream effectors expression of the tyrosine phosphatase SHP2 dephosphorylated KRAS and thereby stimulated RAF/MEK/ERK signaling, suggesting that SHP2 and possibly PTPN2 are important factors in RAS-driven transformation [57,59]. In line with a critical role of SHP2 in the regulation of KRAS, deletion of *Ptpn11* nearly completely blocked the formation of PanIN lesions in *Kras*-driven mouse models [61]. Furthermore, the requirement of *Ptpn11* for PDAC development was also evident in more aggressive mouse models of PDAC (e.g., *Kras^G12D^* and deletion of *Tp53*). SHP099, an allosteric inhibitor that stabilizes an auto-inhibited conformation of SHP2 [62,63], reduced the viability of PDAC organoids and significantly inhibited tumor growth in PDAC xenografts in mice [56]. Recent preclinical studies demonstrated strong synergistic effects between SHP2 and MEK inhibitors (e.g., trametinib) in opposing PDAC and other cancers. These synergistic effects are mediated at least in part by preventing “rebound” re-activation of ERK in response to inhibition of MEK [64,65,66] and upstream compensatory pathway activation as a result of eliminating negative feedback loops mediated by the ERKs [67], as shown in Figure 2. The finding that inhibition of SHP2 keeps mutant KRAS in a phosphorylated (inactive) state suggests an additional mechanism that explains synergistic effects between SHP2 and MEK inhibitors [57]. 

The possibility of deploying SHP2 inhibitors in the treatment of PDAC and other cancers with oncogenic *RAS* mutations is attracting intense interest, and therefore has increased the importance of understanding the precise mechanism(s) by which of SHP2 contributes to signal transduction. Current evidence suggests that SHP2 operates at two levels, as shown in Figure 2. Firstly, as a tyrosine phosphatase that directly acts on RAS family members, favoring the dephosphorylated (active) form of RAS [56,60] and secondly, as a scaffold that facilitates the recruitment of GRB2-SOS to phosphorylated tyrosine residues in receptors and/or scaffolds at the plasma membrane, thereby promoting SOS-mediated GTP-GDP exchange on RAS [68,69]. Thus, SHP2 is emerging as a target that enlarges the repertoire of potential therapeutic strategies against KRAS-mutant tumors [70], principally in combination with MEK inhibitors [64,65,66]. Accordingly, a clinical trial testing an inhibitor of SOS-RAS interaction (BI 1701963) either alone or in combination with the MEK inhibitor trametinib is ongoing in solid tumors with *KRAS* mutation (NCT04111458). However, as described in the next section, several lines of evidence indicate that the most aggressive subtype of advanced PDAC exhibits diminished dependency on oncogenic *KRAS*, underscoring the importance of molecular subtyping of PDAC for identifying participants in future clinical trials as well as prompting the development of additional approaches for its treatment.

## 3. PDAC Subtypes and KRAS Dependency 

Pancreatic cancer is heterogeneous comprising different subtypes, which are not distinguishable by their oncogenic mutations. In an attempt to define different subtypes of the disease, several groups have performed large-scale transcriptomic analyses using resectable tumors and cell lines [71,72,73] leading to the identification of two broad subtypes designated as basal-like (alternatively named squamous or quasi-mesenchymal) and classical-pancreatic, including pancreatic progenitor, immunogenic and exocrine-like subtypes (reviewed in [74]]. The basal-like/squamous subtype is characterized by poor differentiation, down regulation of endoderm specification genes, including GATA binding protein 6 (GATA6) and worse survival (median overall survival of 10–13 months) as compared with 26 months for the more differentiated classical subtype [75]. Several groups are identifying surrogate biomarkers to assess the usefulness of molecular subtyping in the clinical setting [75,76,77]. For example, low GATA6 expression and high Keratin 5 expression correlated with the basal-like subtype and resistance to Folfirinox [76,77,78]. Additional analysis using physical or virtual separation of cell types within the tumors, is subdividing the two major subtypes of PDAC in additional subgroups [79,80]. Recent studies using single-cell and single-nucleus RNA-seq, as well as pancreatic organoids [81], support the notion that the classical and basal-like/squamous PDAC cells coexist in variable proportions in most PDACs (reviewed in [82] and references therein). Accordingly, recent studies showed coexistence of basal-like and classical subtypes within individual PDAC tumors [79,83]. It is important to emphasize that the molecular subtypes of PDAC are not associated with somatic mutations but appear linked to the function of distinct gene regulatory networks and epigenetic events [82]. Thus, the mechanisms driving the generation of PDAC subtypes remain incompletely understood and of importance, because switching basal subtype to classical subtype may provide a strategy to increase sensitivity of PDACs to available therapeutic interventions. 

Interestingly, a recent preclinical study using a set of genetically engineered mouse models induced expression of oncogenic *Kras* and deletion of *Tp53* in either acinar or ductal cell compartments of the pancreas in adult mice [29]. Transcriptomic analyses indicated that the ductal cell–derived tumor signature is enriched in the basal-like/squamous subtype, whereas the acinar cell–derived tumor signature is enriched in the classical subtype. These results imply that cell of origin may be one of the factors that determines subtypes of PDAC [29]. In another study, single-cell suspensions of patient-derived organoids were injected directly into the ducts of the murine pancreas [84]. The resulting lesions could be divided into two main subtypes: a slow-progressing subtype with a glandular phenotype (similar to the classical subtype) and a fast-growing more invasive and less glandular subtype with abundant stromal deposition (similar to the basal-like/squamous subtype). Thus, the results of these studies support the notion that the cell of origin and/or the surrounding microenvironment (rather than genetics) play significant roles in determining PDAC subtype [29,84].

In the context of this article, the precise association of *KRAS* mutation with the molecular subtypes of PDAC is of major interest. Although a critical role of oncogenic *KRAS* in PDAC initiation is established, it appeared that oncogenic *KRAS* was not obligatory for the survival of poorly differentiated PDAC cell lines [85]. Subsequent studies demonstrated that *KRAS* is dispensable for the survival of basal-like/squamous subtype tumors [71,86,87]. For example, Collisson et al. [71] demonstrated that classical PDAC cell lines were more dependent on KRAS than squamous PDAC lines, as shown by siRNA-mediated knockdown of KRAS. A subsequent study using CRISPR (clustered regularly interspaced short palindromic repeats)/Cas-mediated *KRAS* knockout substantiated that endogenous mutated *KRAS* is dispensable for the survival and proliferation of a subset of PDAC cell lines and concluded that basal-like/squamous PDAC is less dependent on oncogenic *KRAS* [86]. Thus, these studies with cells in culture indicated that basal-like/squamous PDAC subtype is relatively independent of oncogenic *KRAS* for survival whereas the classical subtype exhibits marked *KRAS* dependency. 

To define further the role of oncogenic *Kras* in the initiation and maintenance of PDAC, several groups generated mice with inducible *Kras^G12D^* in the pancreas. In these models, the expression of *Kras^G12D^* can be switched on and off by administration or withdrawal of doxycycline [88,89]. Initial experiments using magnetic resonance imaging to follow individual animals in longitudinal studies, confirmed that elimination of *Kras^G12D^* expression resulted in striking tumor regression [88,89]. However, subsequent work showed spontaneous relapse of PDAC following withdrawal of doxycycline in many of mice [90]. While half of the recurrent PDAC re-expressed oncogenic Kras, the other half did not express this oncogene [90], implying that an alternative pathway was driving the recurrence. It is therefore plausible that the contribution of oncogenic KRAS to PDAC initiation can be uncoupled from its contribution to the maintenance of advanced PDAC.

Although intense efforts are being made to target oncogenic KRAS using multiple approaches (reviewed in [11,91]), it is increasingly appreciated that this strategy might not be effective in avoiding relapse or beneficial for the basal-like/squamous subtype of PDAC, the most aggressive subtype of the disease. Consequently, it is of paramount importance to identify the mechanisms and oncogenic drivers that diminish or bypass KRAS dependency, because they could offer key targets in the most aggressive subtype of PDAC. While the mechanisms that potentially drive RAS independence are complex and involve changes in the cancer cells as well as in the tumor microenvironment [92,93], we will focus next on the regulation of YAP/TAZ, as increasing evidence indicates that the activation of these transcriptional co-activators bypasses the need of continued oncogenic RAS signaling in a subset of PDAC. 

## 4. Mechanisms That Circumvent KRAS in PDAC: The Role of YAP/TAZ

The transcriptional co-activators Yes-Associated Protein (YAP) [94] and its paralog WW-domain-containing Transcriptional co-Activator with PDZ-binding motif (TAZ) [95] emerged as fundamental points of convergence and intersection of many signal transduction pathways. These include pathways implicated in the regulation of development, metabolism, organ-size, positional sensing, tissue regeneration, epithelial-mesenchymal transition (EMT), mitosis, micropinocytosis, and adaptation to hypoxia all of which represent fundamental programs for tumorigenesis [53,96,97,98,99,100,101,102]. Here, we will discuss that YAP/TAZ not only cooperates with KRAS in PDAC initiation but also can substitute for KRAS in supporting the survival of the most aggressive subtype of advanced PDAC.

### 4.1. YAP Regulation: Succinct Description

A major regulator of YAP/TAZ activity is the tumor suppressive Hippo pathway [96], which consists of a serine/threonine kinase cascade comprised by the mammalian sterile 20-like kinase 1 or 2 (MST1 or MST2), which binds and phosphorylates the scaffold protein Salvador homolog 1 (SAV1). The active MST–SAV1 complex then phosphorylates and activates the downstream kinases large tumor suppressor homolog 1 and 2 (LATS1 and LATS2) as well as the scaffold proteins MOB kinase activator 1A and 1B (MOB1A and MOB1B) [103]. Activated LATS1/2, in complex with its regulatory protein MOB1, phosphorylates YAP and TAZ, the major downstream effectors of the Hippo pathway and novel sensors of the mevalonate and glycolytic pathways [97,104,105]. Recent studies identified other kinases and scaffolds in the Hippo pathway [106,107,108,109,110], including MAP4K1/2/3 and MAP4K4/6/7, which activate LATS1/2 and thereby lead to YAP/TAZ phosphorylation [106,107,108]. In turn, the proteins of the angiomotin family (e.g., AMOT, AMOTL1 and AMOTL2) stimulate LATS1/2 activation and serve as scaffolds connecting LATS1/2 to both SAV1-MST1 and YAP [109]. Similarly, the WWC proteins function as scaffolds that stimulate LATS1/2 [110]. Clearly, these studies illustrate that Hippo is not a linear pathway but a complex network that regulates YAP/TAZ localization and stability.

YAP and TAZ share nearly half of the overall amino acid sequence, have similar topology and highly conserved residues located within a consensus sequence phosphorylated by LATS1/2 (HXRXXS) but also differ in their gene-regulatory functions [111]. The phosphorylation of YAP by LATS1/2 at Ser^127^ and Ser^397^ (and equivalent residues in TAZ) restricts its cellular localization to the cytoplasm and promotes its protein degradation, respectively. When the Hippo pathway is not functional, YAP and TAZ are dephosphorylated and translocate to the nucleus where they bind and activate a number of transcription factors, primarily the TEA-domain DNA-binding transcription factors (TEAD 1–4). In turn, YAP/TAZ–TEAD complex can interact and functionally collaborate with other DNA-binding partners [112] via promoters and enhancers. 

In this manner, nuclear YAP and TAZ stimulate the expression of multiple genes and display a degree of functional redundancy [113,114] but also differ in a number of ways, including mode of interaction with TEADs [115]. For example, overexpression of YAP negatively regulates TAZ, while YAP knockdown results in increased expression of TAZ. In contrast, TAZ expression levels do not reduce YAP abundance [116]. Results from different laboratories show that the activation of the Hippo network in response to cell/cell contacts, cell polarity and mechanical tension, potently inhibits the transcriptional co-activator activity of YAP and TAZ and promotes TAZ degradation [96,113,117,118,119]. It is important to point out that *YAP1* comprises nine exons and generates at least eight differentially spliced YAP1 isoforms [120], which appear to have different roles in signal transduction [121] adding further levels of complexity.

While the Hippo network is a major mechanism of control of YAP and TAZ activity, recent studies identified additional posttranscriptional modifications that regulate YAP localization and activity in a Hippo pathway-independent manner [122]. These include phosphorylation on serine/threonine residues by multiple other kinases, including AMP-activated protein kinase (AMPK) [123,124], cyclin-dependent kinase 1 (CDK1) [125], cyclin-dependent kinase 7 (CDK7) [126], MAP kinase-activated protein kinase 5 (MK5) [127], MST4 [128] and nemo-like kinase (NLK) [129]. The occurrence and precise significance of these and other modifications of YAP and TAZ in PDAC remain largely unknown and an area that requires further experimental work. In a separate section, we will discuss SFK-mediated YAP tyrosine phosphorylation, a subject of major interest in the context of this article.

### 4.2. Role of YAP in PanIN and PDAC Development and Maintenance

A number of studies indicated that YAP and TAZ are overactive in PDAC patient tumor samples, as judged by expression and/or localization [90,130,131]. Furthermore, recent reports identified YAP expression as an independent prognostic marker for overall survival of PDAC [53,87,132]. Accordingly, we found that multiple YAP/TEAD-regulated genes are associated with unfavorable survival of PDAC patients [53].

In mouse models, *Yap* deletion from the pancreatic epithelium does not interfere with normal pancreatic development or homeostasis [133,134]. In contrast, several studies indicated that Yap is a major effector of Kras-induced PanIN formation [87,133,134]. Zhang et al. found that *Yap* is required for the formation of advanced PanIN lesions and progression to invasive PDAC in mutant *Kras* or *Kras:Trp53* mice [133]. A subsequent study found that deletion of both *Yap* and *Taz* prevented acinar-ductal metaplasia (ADM) after the transient induction of pancreatitis by cerulein administration in KC mice [134], probably by preventing CTGF upregulation [135]. Yap stimulates *Myc* expression and cooperates with this oncogenic transcription factor in the regulation of metabolism [136]. Yap also promotes the immunosuppressive microenvironment in PDAC via recruitment of myeloid-derived suppressor cells [137]. Therefore, extensive evidence indicates that Yap and Taz play a major role downstream of Kras in the initial stages of PanIN formation and PDAC development.

Substantial evidence also indicates that Yap hyper-activation can substitute for Kras function following extinction of *Kras* expression. Using an inducible *Kras^G12D^* mouse model of PDAC described above [88,89], Kapoor et al. showed that a substantial number of recurrent PDAC was driven by overexpression of *Yap* [90]. In parallel studies, Shao et al. performed a genetic screen to identify open reading frames (ORFs) that promote survival of *KRAS*-dependent cancer cell lines following short hairpin RNA (shRNA)-mediated KRAS knockdown [138]. These investigators demonstrated that YAP expression rescued cell viability upon down-regulation of KRAS. Subsequent studies demonstrated that YAP also plays a critical role in promoting macropinocytosis, an important nutrient uptake mechanism also stimulated by KRAS [139]. Collectively, these studies indicate that YAP not only acts downstream of KRAS during PDAC initiation but also that its hyper-activation can circumvent the need of KRAS mutant signaling for PDAC maintenance [90,138,139,140,141]. 

Furthermore, Yap/Taz hyper-activation produced in mice by dual deletion of *Lats1* and *Lats2* to inactivate the Hippo pathway in pancreatic ductal cells induced rapid development of PanIN lesions and subsequently of carcinoma in situ [142]. In contrast, activation of Yap/Taz in acinar cells generated acinar-ductal metaplasia, fibrosis and inflammation but did not induce high-grade PanIN lesions [143]. 

In line with the concept that YAP/TAZ can substitute for oncogenic *KRAS* and thus reduce PDAC dependency on this oncogene, a recent study demonstrated preferential YAP activation in PDAC of the basal-like/squamous subtype [87]. Conversely, forced overexpression of the active YAP^S127A^ mutant in PDAC cells of the classical subtype enhanced their malignant phenotypes and transformed them into squamous subtype while depletion of YAP1 specifically suppressed tumorigenesis of squamous subtype PDAC cells [87]. These investigators also found that WNT5A, a ligand that induces YAP/TAZ activation via non-canonical WNT signaling [144] is overexpressed in PDAC [87,145]. Furthermore, WNT5A substituted for oncogenic *Kras* in tumor maintenance [87]. All these findings support the notion that YAP-mediated signaling contributes to the basal-like/squamous PDAC subtype.

The studies discussed in this section generated with human and mouse cells in culture, genetically modified mice and specimens from PDAC patients, indicate that YAP is a potent pro-oncogenic factor in PDAC and plays a major role in bypassing KRAS function in the advanced basal-like/squamous subtype of the disease, characterized by poor survival. Recent results show that YAP interacts physically and functionally with other key players in PDAC tumorigenesis. These include the Zinc finger E-box-binding homeobox 1 (ZEB1) [146,147,148], the ATP-dependent chromatin remodeling complex SWI/SNF [34,149], the transcription factors of the AP1 family [142]and p53 family members, including p63 isoforms [150]. These interactions further support the notion that YAP/TAZ play a central role in driving PDAC. For example, p63 interacts with Actin Like 6A (ACTL6A), a subunit of SWI/SNF, to decrease the expression of WWC1 [110] which stimulates LATS1/2 and thereby prevents YAP nuclear translocation [151]. Reciprocally, the absence of deubiquitinating enzyme BAP1 reduces the level of LATS1/2 leading to YAP activation and increased PDAC development in KC mice [152]. Accordingly, the expression of either ACTL6A or SMARCA2 (SWI/SNF Related, Matrix Associated, Actin Dependent Regulator of Chromatin, Subfamily A, Member 2), a catalytic subunit of SWI/SNF [153], is associated with unfavorable prognosis while expression of BAP1 is correlated with favorable prognosis in PDAC patients.

## 5. Regulation of YAP by SFK-Mediated Tyrosine Phosphorylation

While YAP plays a critical role in PDAC, mutations in the Hippo pathway components themselves are rare in PDAC, emphasizing the importance of identifying upstream pathways that control YAP/TAZ transcriptional co-activator activity. These include signals mediated by ligand-activated GPCRs, tyrosine kinase and cytokine receptors, integrin-mediated sensing of the extracellular matrix and mechanical cues from the microenvironment. A variety of signaling pathways activated by these receptors, including PI3K, mTOR, PKD, SFK and Rho/actin cytoskeleton converge on the regulation of YAP/TAZ phosphorylation, localization and transcriptional co-activator activity [96,113,117,118,119]. Our own work with human PDAC cells that correspond to the basal-like/squamous sub-type [154] demonstrated that crosstalk between insulin/IGF-1 receptor and GPCR signaling systems [155,156] induces robust nuclear YAP localization, decreases phosphorylation at sites targeted by LATS1/2 and stimulates transcriptional co-activator activity through PI3K and PKD [154]. In many cell types, changes in actin cytoskeletal organization play a critical role in YAP/TAZ activation [112], including in PDAC cells [157].

The SFKs regulate cytoskeletal organization, migration, adhesion and proliferation and are implicated in promoting EMT, invasion and metastasis in multiple tumors [55,158,159,160], including PDAC [161,162]. As mentioned above, multiple autocrine, paracrine, matricrine and yuxtacrine signals stimulate SFK activity [163]. An early study showed that SFK activity is upregulated during progression to invasive PDAC and correlates with survival [164]. A subsequent study using mouse models concluded that Ras/Src cooperate in accelerating PDAC onset and suggested Src-directed therapies in pancreatic cancer [165]. In addition, Src kinase activation is inversely correlated with E-cadherin expression in human PDAC samples (a hallmark of EMT) further implicating the SFKs in EMT [166]. In turn, the EMT program [167] is associated with the basal-like/squamous subtype of PDAC [79].

Importantly, a number of studies in a variety of cell types indicate that SFKs play a major role in the regulation of YAP function through multiple mechanisms, including Hippo-dependent and Hippo-independent pathways [168,169,170,171,172,173], as illustrated in Figure 2. Hippo-dependent regulation of YAP by SKF includes direct phosphorylation of LATS1 by SFK on multiple tyrosine residues that inhibit its catalytic activity and thereby activate YAP/TAZ [168]. Alternatively, SFK phosphorylate and inhibit proteins, such as GPCR-kinase-interacting protein 1 (GIT1), which interact with LATS and repress its activity, thereby enhancing YAP/TAZ activity [169]. SFK also regulate YAP activity via Hippo-independent pathways, including direct phosphorylation of YAP on tyrosine residues, including Tyr^357^ [170,171,172]. Conversely, SHP2 directly downregulates YAP activity by dephosphorylating Tyr^357^ [174]. We recently found that stimulation of human pancreatic cancer cells with a combination of the GPCR agonist neurotensin and insulin induces a marked increase in YAP phosphorylation at Tyr^357^, which was prevented by exposure to SFK inhibitors. Thus, SFK can activate YAP and TAZ through multiple mechanisms in a cell-context dependent manner. Intriguingly, based on the current mechanistic evidence discussed in this and previous sections, SFK and SHP2 exert symmetric opposite effects on YAP and RAS activity (Figure 2). 

## 6. Implications

**Figure 3:**
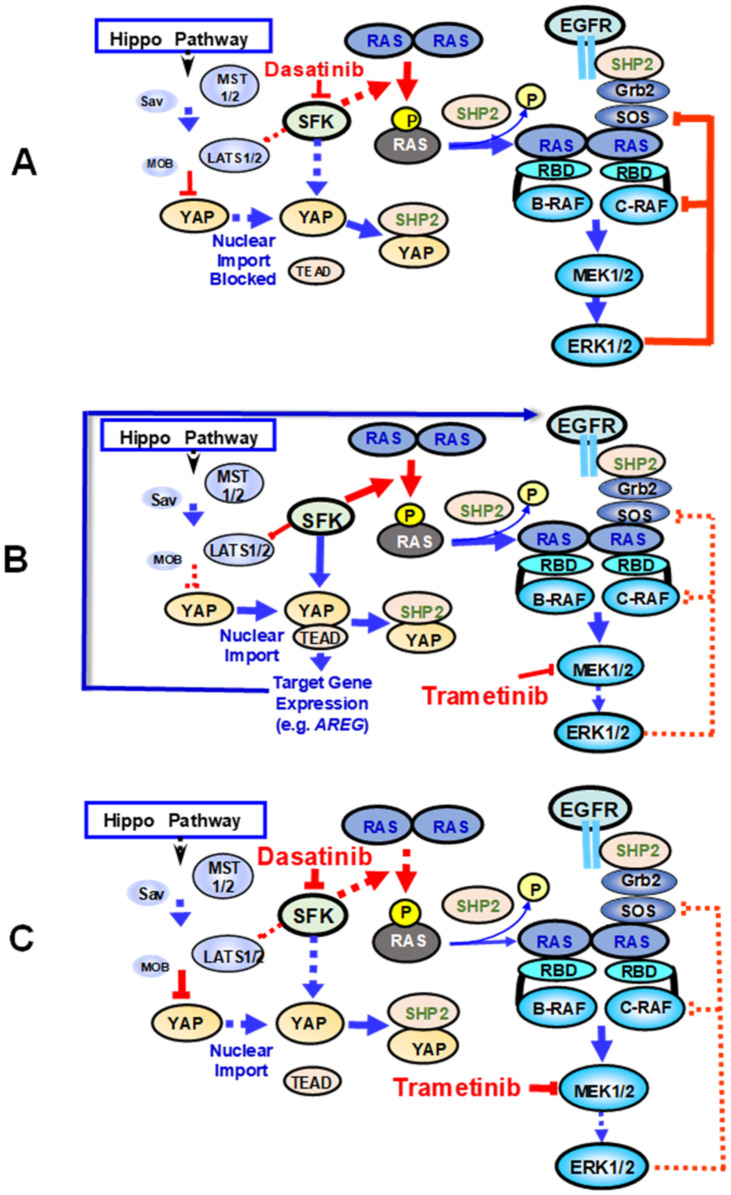
(**A**). Effect of dasatinib on RAS, SKF and YAP crosstalk. Dasatinib, a potent inhibitor of all isoforms of the SFK, prevents YAP activation but removes inhibitory phosphorylation of KRAS, leading to hyper-activation of RAF/MEK/ERK, thus providing an escape route from the SKF block. (**B**) Effect of trametinib on RAS, SKF and YAP crosstalk. Trametinib, a potent MEK inhibitor, releases feedback loops leading to compensatory activation of upstream SOS leading to recovery of MEK activity. SHP inhibitors block KRAS reactivation in trametinib-treated cells but could induce YAP activation by preventing its de-phosphorylation. (**C**) Effect of dasatinib and trametinib on RAS, SKF and YAP crosstalk. Combination of drugs such as dasatinib and trametinib acting at different points of the crosstalk between RAS, SFK and YAP are likely to prevent drug resistance caused by inhibition of compensatory feedback loops. FDA-approved inhibitors of EGFR (e.g., efitinib, erlotinib, afatinib, and osimertinib) and RAF kinases could also be used to prevent their over-activation in response to MEK inhibition (omitted from the Figure for clarity). Please also see the main text for further explanation.

The findings discussed here are not only highly relevant for understanding new aspects of PDAC biology but also have important implications for PDAC therapy. We anticipate that monotherapy targeting SFK is not going to be successful because SFK inhibitors will remove inhibitory effects on RAS, leading to its hyper-activation, thus providing an escape route through RAF/MEK/ERK (Figure 3A). In line with this notion, dasatinib diminished metastatic dissemination but did not interfere with the growth of the primary tumor in a preclinical model of PDAC [164]. Furthermore, dasatinib with gemcitabine [175,176] or 5-fluorouracil and oxaliplatin [177] failed to demonstrate significant clinical benefit in separate clinical trials. Similarly, single-agent MEK inhibition was ineffective [178] in most cases, possibly because it releases feedback loops leading to compensatory activation of upstream or parallel pathways [67], as indicated in Figure 3B. Therefore, we suggest that inhibition of both SFK and MEK/ERK to block YAP and curtail RAS hyper-activation could provide a plausible approach that warrants further mechanistic and clinical studies (Figure 3C). In support of this notion, combinations of SFK and MEK inhibitors exhibited synergistic effects on PDAC patient-derived cell lines and xenografts [179] and other KRAS-mutant cancer models both in vitro and in vivo [180]. In addition, we found that SFK and MEK inhibitors exhibit potent synergistic effects inhibiting colony formation by human PDAC cells. As described before, there is great deal of interest in targeting SHP2 to prevent “rebound” MEK/ERK activation in the setting of trametinib administration [64,65,66]. However, inhibition of SHP2 could increase tyrosine phosphorylation of YAP [174], and thus lead to drug resistance via YAP activation, another escape route from the SHP2/MEK block (Figure 3). It follows that strategies using targeted therapies for PDAC should take into consideration the molecular subtype and the newly identified crosstalk between RAS, SFK and YAP in designing drug combinations that overcome activation of compensatory pathways leading to drug resistance.

## 7. Conclusions

Overwhelming evidence substantiates the crucial significance of KRAS mutations in PDAC development. Consequently, the development of new drugs that target RAS or pathways upstream and/or downstream of RAS remains a major effort. Inhibition of RAS dimerization, as exemplified in Figure 1, may offer a new approach in this direction. Although intense academic and pharmaceutical attempts are being made to target oncogenic KRAS using multiple approaches (reviewed in [11,91]), this therapeutic intervention might not be effective to avoid relapse of the disease or target advanced PDAC of the basal-like/squamous subtype, the most aggressive subtype of PDAC.

In this framework, recent studies identified opposite effects of SFK-mediated phosphorylation on KRAS and YAP function—namely, inhibition of KRAS signaling by phosphorylation on Tyr^32^ and Tyr^64^ and activation of YAP via Hippo-dependent or Hippo-independent pathways, including direct phosphorylation at Tyr^357^ (Figure 2). These findings are surprising and imply that either SFK functions in a tumor-suppressive capacity [56] or that continued RAS signaling might not be crucial in at least certain subtypes of PDAC in which the SFK/YAP axis plays a prominent role. We hypothesize here that these contrasting effects of SFK on KRAS and YAP/TAZ contribute to the emergence of basal-like/squamous PDAC, the PDAC subtype that displays diminished dependence on KRAS. In this framework, the active SFK/YAP axis propels PDAC cells to a state of high metastasis, epithelial-mesenchymal transition and reduced dependence on KRAS signaling, salient features of the basal-type/squamous subtype of PDAC. 

Since extracellular signals regulate SFK activation in a dynamic and reversible manner [163], the YAP-dependent and KRAS-independent state, characteristic of basal-like PDAC, is reversible and responds to extracellular signals, including adjacent pancreatic cells. We envisage that the opposite effects of SFK on KRAS and YAP provide a flexible mechanism that enables PDAC cells to initiate EMT and metastasis when SFK/YAP signaling predominates and explain coexistence of different subtypes of PDAC in different locations of the same tumor. Indeed, well-differentiated and poorly differentiated (basal-like/squamous) PDAC cells coexist in variable proportions in most PDACs [82].
